# Skeleton Segmentation on Bone Scintigraphy for BSI Computation

**DOI:** 10.3390/diagnostics13132302

**Published:** 2023-07-06

**Authors:** Po-Nien Yu, Yung-Chi Lai, Yi-You Chen, Da-Chuan Cheng

**Affiliations:** 1 Department of Biomedical Imaging and Radiological Science, China Medical University, Taichung 404, Taiwan; lu09200016@gmail.com (P.-N.Y.); nses123789@gmail.com (Y.-Y.C.); 2 Department of Nuclear Medicine, Feng Yuan Hospital Ministry of Health and Welfare, Taichung 420, Taiwan; daniellai999@hotmail.com; 3 Center of Augmented Intelligence in Healthcare, China Medical University Hospital, Taichung 404, Taiwan

**Keywords:** Mask R-CNN, Double U-Net, Deeplabv3 +, bone segmentation, bone scintigraphy

## Abstract

Bone Scan Index (BSI) is an image biomarker for quantifying bone metastasis of cancers. To compute BSI, not only the hotspots (metastasis) but also the bones have to be segmented. Most related research focus on binary classification in bone scintigraphy: having metastasis or none. Rare studies focus on pixel-wise segmentation. This study compares three advanced convolutional neural network (CNN) based models to explore bone segmentation on a dataset in-house. The best model is Mask R-CNN, which reaches the precision, sensitivity, and F1-score: 0.93, 0.87, 0.90 for prostate cancer patients and 0.92, 0.86, and 0.88 for breast cancer patients, respectively. The results are the average of 10-fold cross-validation, which reveals the reliability of clinical use on bone segmentation.

## 1. Introduction

Bone is the most common targeted site for metastatic cancer, especially in the advanced and later phases of cancer progression—notably breast, prostate, and lung cancers, with the highest incidence rates [1]. Bone metastases can severely impact patients’ daily activities and quality of life due to severe pain and associated major complications. The protracted clinical course of bone metastasis poses significant challenges to treatment. Per a 2022 report published in the Taiwan National Health Insurance Research Database [2], prostate cancer ranked sixth among the leading causes of cancer death among Taiwanese men. In contrast, breast cancer ranked second among the leading causes of cancer death among Taiwanese women. Diagnostic techniques for bone metastasis currently include bone scintigraphy (BS), X-ray imaging, computed tomography (CT), and magnetic resonance imaging (MRI), while BS serves as the most cost-effective early screening method. BS can diagnose bone metastasis earlier than CT or X-ray, within 3 to 6 months [3].

Bone metastasis typically affects the central skeletal system and the proximal regions of the upper and lower limbs. The central skeletal system contains red bone marrow, which may contribute to the formation of bone metastasis due to its physiological characteristics [4]. Physicians often perform a whole-body bone scan (WBBS) to diagnose the presence of bone metastasis. ^99m^Tc-MDP is the radiopharmaceutical injected into a patient’s vein, which can enter the bone cells and deposit with mineral components in four hours. Consequently, Tc-99m MDP tends to accumulate in areas of active bone formation in the affected region, resulting in localized increased radiopharmaceutical activity that appears as a “hot spot” on BS, allowing physicians to identify bone metastasis [5]. However, BS may suffer from ambiguity owing to impacts such as bone injury, arthritis, and degenerative changes, and causes interpretation challenges. Inexperienced clinical physicians may struggle to make accurate judgments or even misinterpret images.

**Figure 1:**
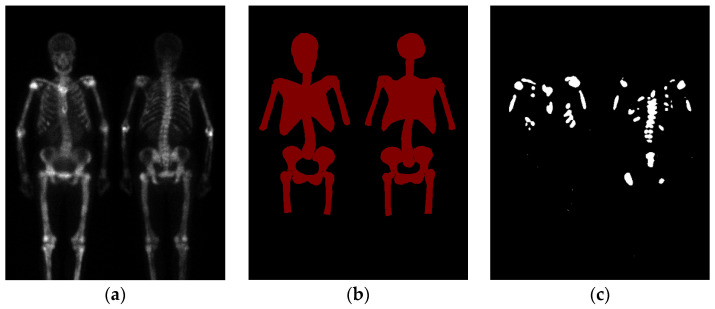
(**a**) represents a WBBS, (**b**) depicts the skeleton regions with a high incidence of bone metastasis, and (**c**) indicates the areas where bone metastasis is present. The BSI in (**a**) corresponds to the area ratio of (**c**) to (**b**).

Bone scan index (BSI) is an imaging biomarker used to quantify the extent of bone metastasis in cancers [6]. BSI is calculated as the ratio of “the number of bone lesions indicating bone metastasis” to “the number of regions with a high incidence of bone metastasis” [7,8,9], as shown in Figure 1. With artificial intelligence, machine learning, and big data, BSI calculation has become more objective, accurate, and diagnostically efficient. BSI’s most attractive application is monitoring treatment and prognosis, providing significant clinical value. Armstrong et al. from Duke University introduced the automated bone scan index (aBSI) as an objective imaging parameter [10], which can evaluate the prognosis of metastatic castration-resistant prostate cancer (mCRPC) patients undergoing systemic treatment in clinical trials. In [11,12], manual and automated BSI measurements were highly correlated (ρ = 0.80), and automated BSI scoring demonstrated reproducibility, eliminating the subjectivity of clinical judgment while retaining the same clinical significance as manual BSI scoring. Furthermore, some studies confirmed the utility of aBSI in mCRPC patients [13,14,15], while other studies have begun to explore its application and refinement in other tumors [16].

Generally, computer-assisted diagnosis (CAD) systems that utilize machine learning or neural network (NN) framework for calculating BSI on WBBS images can be divided into two parts: lesion segmentation and skeleton segmentation, which respectively reflect the numerator and denominator of the BSI value [17,18,19,20]. Recently, numerous studies [21,22] and related patents [23,24] on lesion segmentation using the NN framework have been conducted. However, the performance of the lesion pixel-wise segmentation has not been thoroughly and rigorously investigated. Similarly, research on skeleton segmentation using deep learning and NN models is scarce in previous studies [20,25] despite the mention of its skeleton segmentation approach in [20], which lacks comparison with other NN models. Although [25] compared its performance with U-Net, it remained confined to traditional semantic segmentation network architectures. Thus, the field of skeleton segmentation using NN remains insufficiently explored. This paper uses different NN models for skeleton segmentation on WBBS images and investigates their results. Additionally, we have built a website platform for online skeleton segmentation of WBBS images [Appendix A], which provides effective skeleton segmentation data for further evaluation of BSI.

## 2. Materials and Methods

### 2.1. Materials

In this retrospective study in collaboration with the Department of Nuclear Medicine at China Medical University Hospital, 196 WBBS images of patients with prostate cancer were collected. Among the 196 patients, 110 patients had bone metastasis, and 86 patients had no evidence of bone metastasis. We also collected 163 WBBS images of patients with breast cancer. All of them had bone metastasis. The study was approved by the Institutional Review Board (IRB) and the Hospital Research Ethics Committee (CMUH106-REC2-130) of China Medical University.

The radiopharmaceutical used for WBBS was Tc-99m MDP, and the imaging was performed 4 h after the vein injection. A Gamma camera (Millennium MG, Infinia Hawkeye 4, or Discovery NM/CT 670 system; GE Healthcare, Waukesha, WI, USA) was used for planar bone scanning, with a low-energy high-resolution or general-purpose collimator, a matrix size of 1024 × 256, a photon energy centered on the 140 keV peaks, and a symmetric 20% energy window. The collected bone scan images were in DICOM format, with a spatial resolution of 1024 × 512 pixels (composed of anterior-posterior (AP) and posterior-anterior (PA) views), and the intensity information of each pixel was saved in 2-byte (uint16). The images were preprocessed using the dedicated GE Xeleris workstation (GE Medical Systems, Haifa, Israel; version 3.1) before being uploaded to PACS.

**Figure 2:**
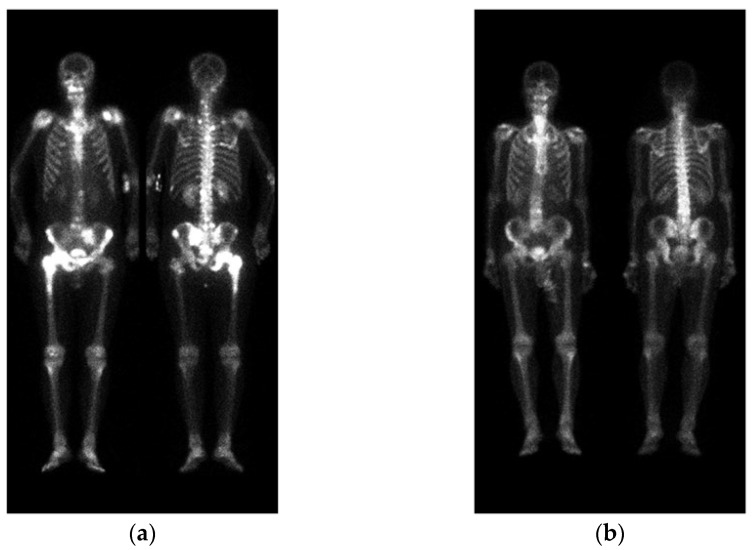
Two WBBS, (**a**) has bone metastasis and (**b**) has no metastasis.

A standard WBBS image contains two views: anterior and posterior. The original DICOM images were first converted to PNG format after removing any identifiable information. Following the approach described in [22], pre-processing was performed by normalizing the image size and intensity. Afterwards, the anterior and posterior views were cropped into a single image with a size of 950 × 512, without any scaling or geometric transformations, as shown in Figure 2.

### 2.2. Region Definition

To identify the skeletal regions where bone metastases occur most frequently, we consulted with two experienced nuclear medicine physicians and established standards. The standards require the approval of these two board-certified nuclear medicine physicians. The regions are the skull, spine, chest (including ribs, scapula, and clavicle), humerus (proximal to midshaft of the femurs), femurs (proximal to midshaft of the humerus), and pelvis.

The positions of the humerus on images differ significantly, as shown in Figure 2. Different from only one category on femurs, we categorize humerus into four categories, i.e., the left and right humerus in the anterior and posterior views separately. The reason for doing so will be addressed in the discussion. Furthermore, Tc-99m MDP undergoes renal metabolism, which can result in the kidneys appearing as high signal areas. In some situations, the kidney will be misclassified as metastasis. To alleviate this problem, we created an extra kidney category to exclude ambiguity.

**Figure 3:**
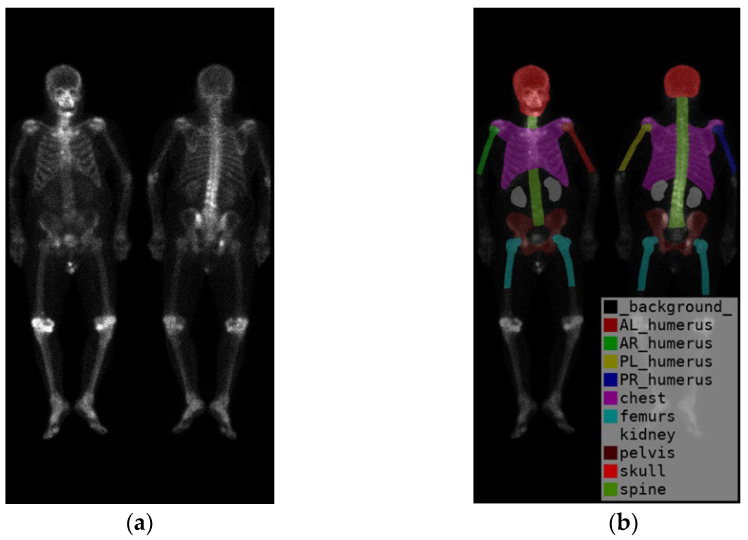
(**a**) shows the original image, while (**b**) displays the ground truth of the bone metastasis-prone regions and 10 (+1 background) categories with different colors.

In summary, there are in total ten categories (Figure 3), including the skull, spine, chest (including ribs, scapula, and clavicle), anterior right humerus (AR), anterior left humerus (AL), posterior right humerus (PR), posterior left humerus (PL), femurs (proximal to midshaft of the humerus), pelvis, and kidney.

### 2.3. Neural Network Architectures

Three different neural network architectures were tested, including Mask R-CNN [26], Double U-Net [27], and Deeplabv3 plus [28]. We used similar hyperparameters on these three models to conduct experiments to compare their performances.

**Figure 4:**
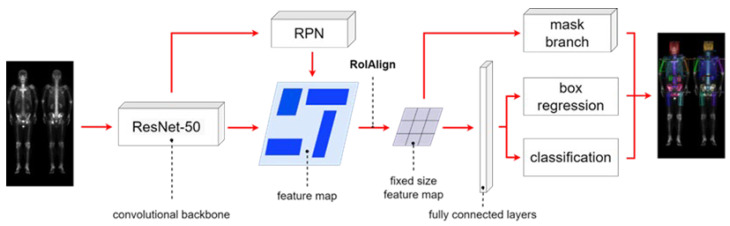
The architecture of Mask R-CNN having multi-class classification.

The Mask R-CNN architecture shown in Figure 4 comprises four main parts: backbone architecture, RPN, RoIAlign, and head architecture. We used ResNet-50 as the backbone. The hyperparameters hold the same learning rate of 0.005, batch size of 4, and 100 epochs.

**Figure 5:**
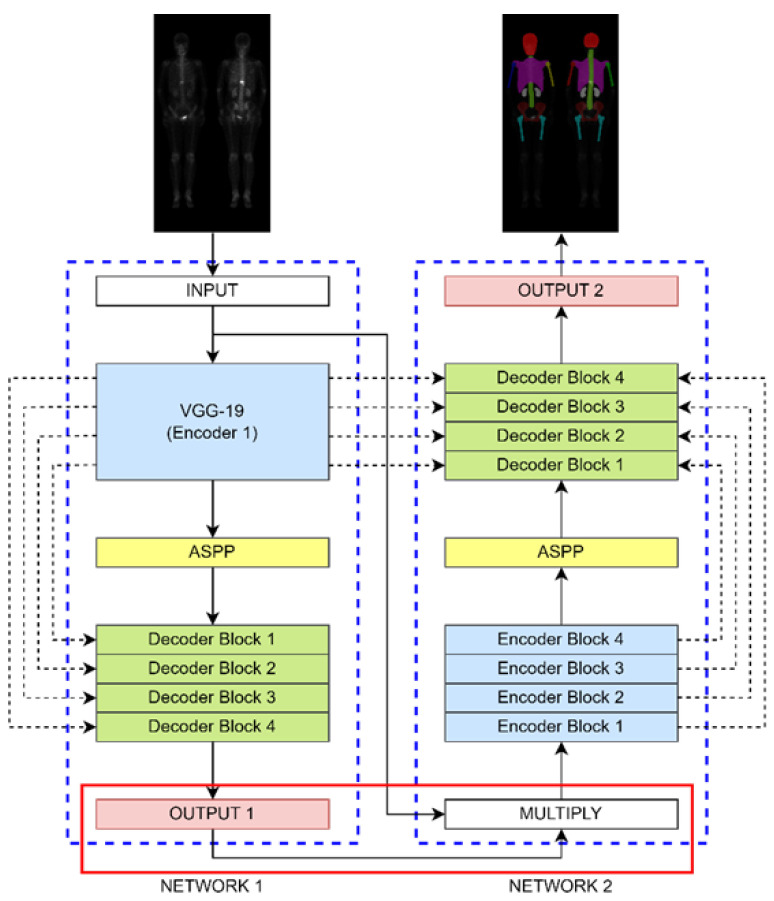
The architecture of Double U-Net is comprised of two sub-networks. To enable multi-class classification, we modified the output of Network 1 and the input of Network 2.

The Double U-Net architecture shown in Figure 5 comprises two sub-networks, dilated convolution, spatial pyramid pooling, and an SE block. It was originally designed for binary classification. Here we modified it to make the multi-class classification. We changed the output layer of Network 1 to have a SoftMax activation function to enable multi-class classification. The hyperparameters were set to be a learning rate of 0.0005, batch size of 4, 200 epochs (without data augmentation), or 20 epochs (with data augmentation).

**Figure 6:**
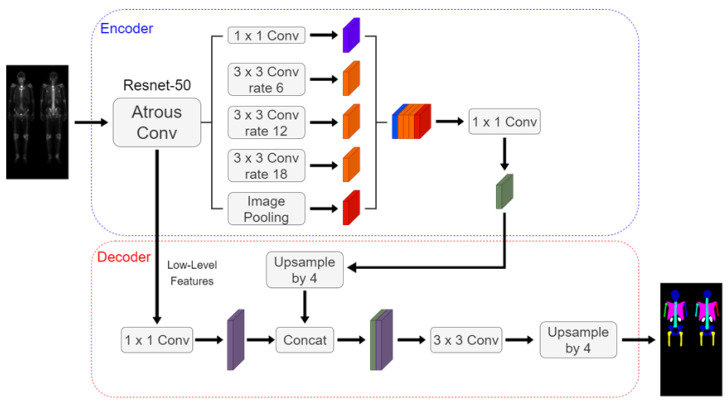
The architecture of the DeeplabV3 plus using ResNet-50 as the backbone.

The Deeplabv3 plus architecture shown in Figure 6 includes an encoder, decoder, dilated convolution, and depth-wise separable convolution. We used ResNet-50 as the encoder backbone. The hyperparameters were set to be a learning rate 0.0005, batch size of 4, and 200 epochs.

The learning rate is a hyperparameter used in various machine learning algorithms, particularly in gradient-based optimization. It determines the step size at which the model updates its parameters during training. The choice of learning rate depends on the specific problem. Typically, every model has its own suggested learning rate. In this study, we choose a balance between accuracy and training speed. For this task, Mask R-CNN uses a learning rate of 0.005, while Double U-Net and DeeplabV3 plus use a learning rate 0.0005.

### 2.4. Image Pre-Processing

The input matrix size for Mask R-CNN was 950 × 512. Double U-Net and Deeplabv3 plus’s input matrix size was adjusted to 960 × 512 by padding with zeros due to their restriction. The labels were saved in PNG format with integers ranging from 0 to 10.

Augmentation included rotations (−3°, 0°, 3°) with step 1°, scaling (0.9, 1, 1.1) with step 0.1, and brightness adjustments (0.8, 0.93, 1.06, 1.19, 1.32, 1.45, 1.58, 1.7 times). The augmented images had the same matrix size as the original images, resulting in a total rate of 63 times increase. The augmentations were only used in training.

### 2.5. Evaluations

In this study, the terms true positive (TP), false positive (FP), true negative (TN), and false negative (FN) were defined in pixel scale. The evaluation metrics used in the experiment were precision (Equation (1)) and sensitivity (Equation (2)), and the overall model evaluation was based on the F1 score (Equation (3)).

Precision = (True positive)/(True positive + False positive),(1)

Sensitivity = (True positive)/(True positive + False negative),(2)

F1 score = 2(Precision × Sensitivity)/(Precision+ Sensitivity),(3)

## 3. Results

### 3.1. 10-Fold Cross-Validation

**Figure 7:**
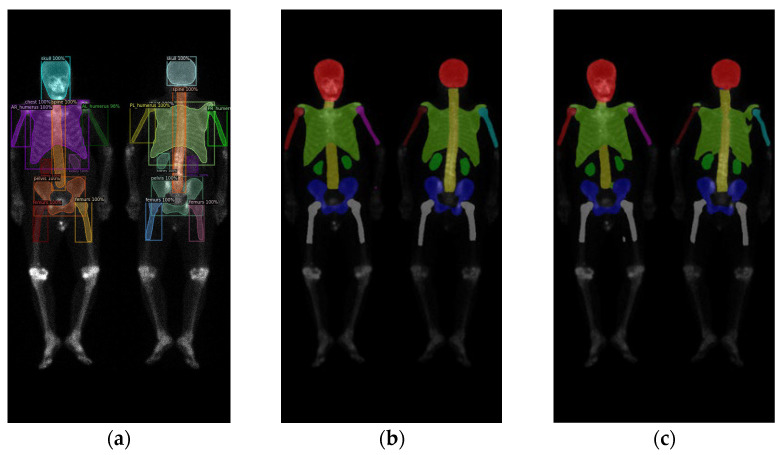
The qualitative results of three models on prostate cancer WBBS images: (**a**) Mask R-CNN, (**b**) Double U-Net, and (**c**) DeeplabV3 plus.

**Figure 8:**
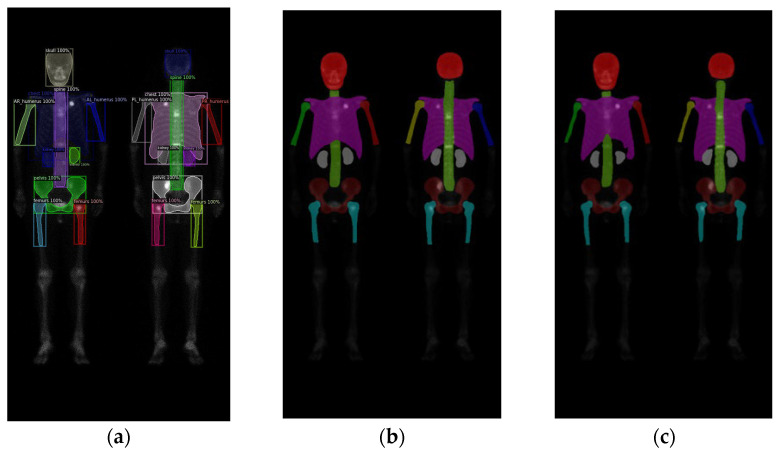
The qualitative results of three models on breast cancer WBBS images: (**a**) Mask R-CNN, (**b**) Double U-Net, and (**c**) DeeplabV3 plus.

In this study, validations on these three models used 10-fold cross-validation. Two datasets comprised 196 prostate cancer WBBS images and 163 breast cancer WBBS images, respectively. The ratio of training, validation, and test was set to be 8:1:1. The main goal of this experiment was to compare the performance differences among each network and to evaluate the impact of prostate and breast cancer WBBS images on network training. The hyperparameters used in the experiment are in Table 1, and the results are depicted in Table 2 and Table 3, compared in Table 4. The qualitative results of bone segmentation are shown in Figure 7 and Figure 8.

### 3.2. 10-Fold Cross-Validation with Data Augmentation

After the above experiments, we chose Double U-Net for investigation because it slightly outperformed on F1-score. Following, we fine-tuned the epoch to trade-off the training time and the performance to see what best performance we could reach. The images of training for prostate cancer and breast cancer were augmented 63 times by using rotation, scaling, and brightness adjustment described in the methods. Again, the hyperparameters are in Table 5, and the quantitative results of the 10-fold cross-validation are in Table 6.

## 4. Discussion

This study utilised Mask R-CNN, Double U-Net, and DeeplabV3 plus for skeleton segmentation comparison on prostate cancer and breast cancer WBBS images. The quantitative results were investigated via 10-fold cross-validation. Based on the quantitative findings, Mask R-CNN exhibited higher precision than Double U-Net by 2.03% in the prostate cancer dataset and 1.84% in the breast cancer dataset. Mask R-CNN also exhibited higher precision than DeeplabV3 by 3.23% in the prostate dataset and 2.31% in the breast dataset. On the other hand, Double U-Net (90.70% & 88.86%) demonstrated higher sensitivity than Mask R-CNN (87.02% & 85.51%) and DeeplabV3 plus (88.64% & 85.71%). This indicated that Mask R-CNN had lower false positives (FP) during prediction, while Double U-Net had lower false negatives (FN).

**Figure 9:**
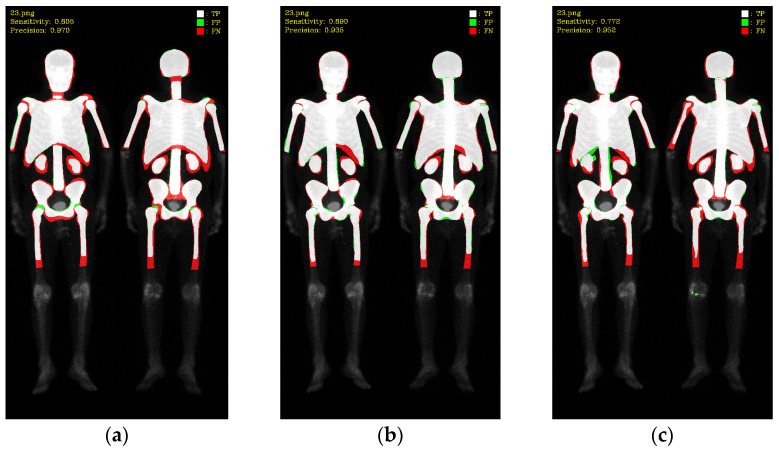
Qualitative comparisons on three models: (**a**) Mask R-CNN, (**b**) Double U-Net, (**c**) DeeplabV3 plus. White: TP, red: FP, and green: FN.

**Figure 10:**
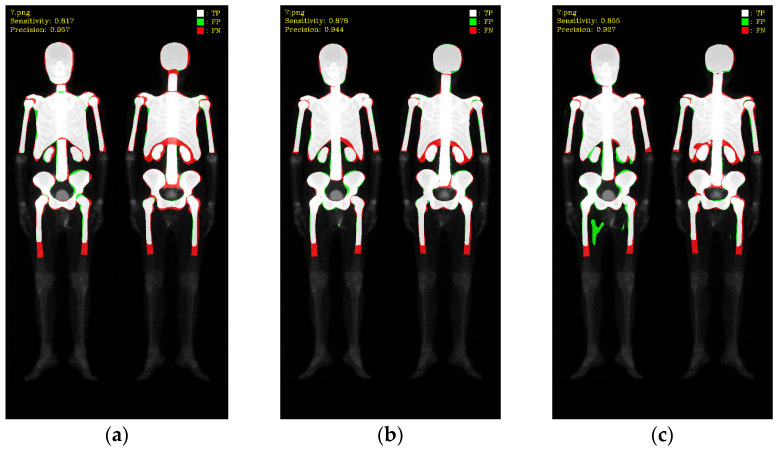
Qualitative comparisons on three models: (**a**) Mask R-CNN, (**b**) Double U-Net, (**c**) DeeplabV3 plus.

To better understand these results, we visualized the predictions, where white color represented TP, green color represented FP and red color represented FN (as shown in Figure 9 and Figure 10). Mask R-CNN’s predictions shifted inward slightly compared to the ground truth (GT), resulting in more FN in the edge regions and only a few FP. Double U-Net’s predictions aligned well with the GT along the edges, resulting in slightly fewer FN but more FP. DeeplabV3 plus exhibited irregularities along the edges compared to the other two models, leading to noticeable erroneous FP and an overall increase in FP.

These findings shed light on the performance of different models for skeleton segmentation, emphasizing the trade-off between FP and FN. Further improvements can be explored to address the limitations observed, particularly in the case of DeeplabV3 plus, to enhance its stability and accuracy.

**Figure 11:**
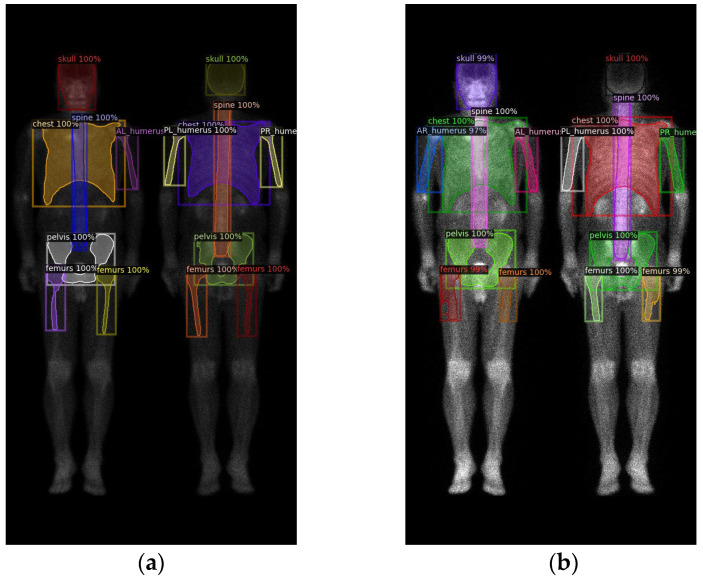
(**a**) Original test segmentation result with missing right humerus in the frontal view. (**b**) Segmentation result after adjusting the brightness to 2.5 times and retest.

Further investigation of Mask R-CNN results revealed an increase in false negatives (FN) when predicting smaller categories, such as the humerus and kidneys, as shown in Figure 11a. This result could be caused by the following reasons:

First, the insufficient brightness in the WBBS image may hinder feature detection. The brightness of WBBS images depends on the counts collected by the scintillation crystal, which can be influenced by factors such as patient thickness and radiopharmaceutical activity. In cases where the received counts are insufficient, resulting in inadequate image brightness, deep neural network models may struggle to make accurate judgments or even make errors. Adjusting the image brightness and conducting further tests can help alleviate this situation, as shown in Figure 11b.

**Figure 12:**
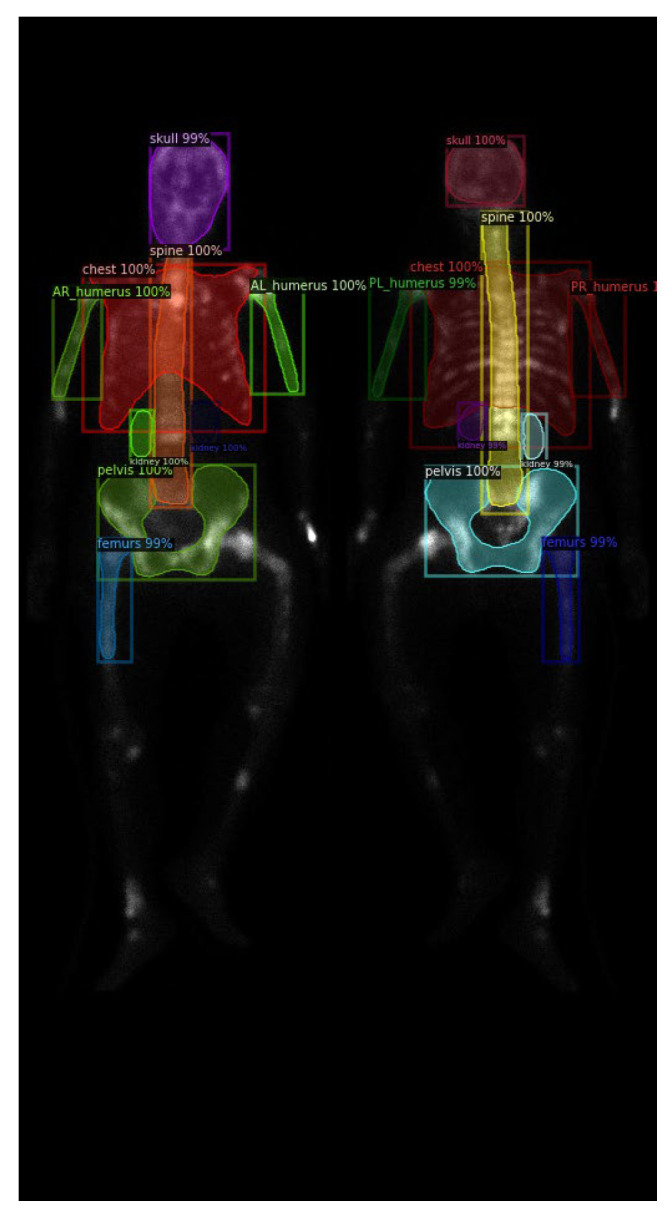
A segmentation result showed the absence of the frontal and dorsal left femur due to abnormal patient position, while this abnormal position was rare and did not exist in the training dataset.

Second, abnormal patient positioning in the WBBS image could cause another issue. In a few instances, patient positioning in the WBBS image deviates to some extent from standard clinical positions. This deviation made challenges for CNN prediction, as shown in Figure 12. The degree of deviation is closely related to the patient’s clinical condition and is difficult to entirely avoid in clinical practice. While other previous studies might manually exclude misleading images to prevent such occurrences, this study aimed to maintain a dataset that reflects real clinical scenarios, thereby we did not exclude any case. To enhance the network’s ability to predict WBBS images with unusual positioning, future considerations include employing hard negative mining techniques to improve the model’s generalization capabilities.

Third, the model’s insensitivity to features of small objects in WBBS images could also decrease performance. Quantitative results indicated relatively low precision for categories such as upper limbs, femurs, and kidneys, which correspond to smaller objects. This suggested Mask R-CNN facing certain difficulties in segmenting smaller regions.

These findings highlighted specific challenges encountered during the skeleton segmentation process, particularly related to image brightness, abnormal patient positioning, and the segmentation of smaller objects. Addressing these challenges could improve the performance of the Mask R-CNN model.

**Figure 13:**
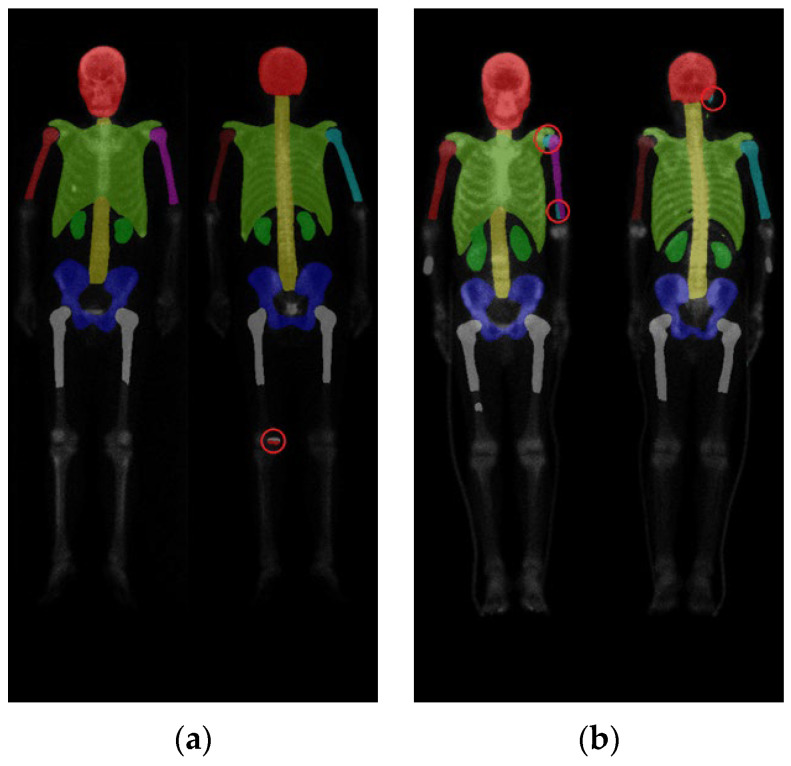
(**a**) Segmentation result of Double U-Net with a segmentation error in the distal part of the leg. (**b**) Segmentation result of Deeplabv3 plus showing category confusion in the upper limbs and head region.

On the other hand, we observed that DeeplabV3 plus and Double U-Net tended to mix categories, resulting in unstable performance. Double U-Net and DeeplabV3 plus did not exhibit the category missing issue observed in Mask R-CNN, but they experienced problems such as category confusion and masks appearing in unintended areas, with DeeplabV3 plus being particularly affected. The issue of category confusion during prediction in semantic segmentation network architectures was not explicitly mentioned in [20,25]. However, in our experiments, we did observe this problem. Figure 13a showed an incorrect segmentation in the knee area in a Double U-Net skeleton segmentation result, while Figure 13b depicted category confusion in the upper limbs and head in a DeeplabV3 plus skeleton segmentation result.

This problem stemmed from different network architectures. Mask R-CNN utilizes parallel branch networks to independently determine categories and select the appropriate masks based on individual region-of-interest (ROI). Consequently, different ROIs could be distinguished independently, and masks could be treated as separate entities. In contrast, traditional fully convolutional network (FCN) architectures performed category and mask predictions simultaneously, leading to competition between different categories and masks. Additionally, due to the design of having one category per mask, FCN-based methods could not treat ROIs independently. Another critical factor was using the Sigmoid activation function and average binary cross-entropy loss in the branch networks, which mitigated the adverse effects of cross-category competition encountered in traditional FCN methods. This design yielded excellent instance segmentation results and avoided category overlap or confusion. From the experiments, Mask R-CNN demonstrated itself more suitable for skeleton segmentation in WBBS images than the other two network architectures.

From experiments shown in Table 2, Table 3 and Table 4, one might think that the models’ performance is close to each other, and there might not be a statistically significant difference. It is crucial to consider the context of image segmentation in deep learning. In this task, precision and sensitivity are calculated pixel-wise. Therefore, even a small difference in percentage points can have a significant impact.

In the experiments involving data augmentation, it was observed that it contributed to a slight performance improvement. As the model already performed reasonably well without data augmentation, the addition of data augmentation only led to marginal performance gains. According to related literature [29], incorporating data augmentation helped reduce overfitting at higher learning rates, allowing the model to be trained for more epochs without sacrificing accuracy. Further experiments and investigations were warranted to explore the impact of data augmentation in more depth.

The limitation of this study is the scarcity of original data and the homogeneity of its source. In the future, it is desirable to establish collaborations with other medical centers to acquire cross-centre data, thereby improving the performance and generalization ability of the models. Additionally, we only investigated three relatively common network architectures, and it would be an attractive research direction to explore newer architectures, such as transformer-based networks. Different nuclear medicine imaging modalities, such as planar and SPECT, differ in the resulting images. It would be worth investigating whether these differences lead to heterogeneity in model predictions. This is an area for further exploration in the future.

## 5. Conclusions

In this study, we investigated three CNN models on bone segmentation of the WBBS images. We found that only one model was suitable for this goal, Mask R-CNN. The Double U-Net and Deeplabv3 + had a problem with ‘category confusion’, which humans would never have. We used a pixelwise scale to examine the model performance. The best performance we had ever made for Mask R-CNN was the precision, sensitivity, and F1-score: 0.93, 0.87, 0.90 for the prostate cancer dataset and 0.92, 0.86, 0.88 for the breast cancer dataset, which was the average of 10-fold cross-validation.

## Figures and Tables

**Table 1:** Hyperparameters were used for the 10-fold cross-validation experiments with each neural network.

Hyperparameters	Mask R-CNN	Double U-Net	DeeplabV3 Plus
Learning Rate	0.005	0.0005	0.0005
Batch Size	4	4	4
Epochs	100	200	200

**Table 2:** The comparing results of 10-fold cross-validation on prostate cancer WBBS image dataset.

Category	Mask R-CNN		Double U-Net		DeeplabV3 Plus	
	Precision	Sensitivity	Precision	Sensitivity	Precision	Sensitivity
Skull	97.22	94.43	96.05	96.13	95.34	95.91
Spine	93.90	88.62	91.16	91.30	89.94	89.79
Chest	95.33	93.58	94.83	94.52	93.61	93.87
AR_humerus	91.82	84.80	89.65	90.18	87.42	87.88
AL_humerus	92.46	85.30	89.76	90.12	87.94	89.02
PR_humerus	91.72	84.41	88.68	89.55	85.77	87.25
PL_humerus	89.94	82.01	87.89	88.78	87.50	83.64
Pelvis	92.32	88.26	90.76	90.83	90.99	87.84
Femurs	88.40	81.75	86.08	84.85	85.59	82.60
Kidney	86.13	79.23	82.45	82.73	80.15	81.87
Average	91.93	86.24	89.73	89.90	88.43	87.97
Average (w/o kidney)	92.57	87.02	90.54	90.70	89.34	88.64

The F1 scores are 89.71, 90.62, and 88.99 for Mask R-CNN, Double U-Net, and DeeplabV3, respectively. The Double U-Net has the best F1-score.

**Table 3:** The comparing results of 10-fold cross-validation on the breast cancer WBBS image dataset.

Category	Mask_R-CNN		Double U-Net		DeeplabV3 Plus	
	Precision	Sensitivity	Precision	Sensitivity	Precision	Sensitivity
Skull	97.24	94.23	96.18	95.88	95.91	93.24
Spine	93.20	88.61	91.15	90.68	90.56	87.76
Chest	95.17	93.48	94.10	94.32	92.78	93.40
AR_humerus	89.67	80.23	87.21	86.01	85.88	81.90
AL_humerus	89.07	81.20	86.44	84.97	87.15	80.26
PR_humerus	89.65	82.10	87.58	86.46	85.41	83.66
PL_humerus	88.28	80.08	87.34	86.39	86.62	81.92
Pelvis	92.22	88.27	90.86	90.24	91.34	87.54
Femurs	89.95	81.39	87.05	84.83	88.06	81.71
Kidney	87.21	80.71	84.37	83.74	83.91	77.59
Average	91.17	85.03	89.23	88.35	88.76	84.90
Average (w/o Kidney)	91.61	85.51	89.77	88.86	89.30	85.71

The F1-scores are 88.45, 89.31, and 87.47 for Mask R-CNN, Double U-Net, and DeeplabV3, respectively. The Double U-Net has the best F1-score.

**Table 4:** The comparing results of 10-fold cross-validation on the two image datasets.

Database	Mask_R-CNN			Double U-Net			DeeplabV3 Plus		
	Pre.	Sen.	F1-Score	Pre.	Sen.	F1-Score	Pre.	Sen.	F1-Score
Prostate cancer	92.57	87.02	89.71	90.54	90.70	90.62	89.34	88.64	88.99
Breast cancer	91.61	85.51	88.45	89.77	88.86	89.31	89.30	85.71	87.47

Pre. = Precision, Sen. = Sensitivity.

**Table 5:** Hyperparameters for training Double U-Net.

Hyperparameters	Double U-Net
Learning Rate	0.0005
Batch Size	4
Epochs	20

**Table 6:** 10-fold cross-validation on Double U-Net, used augmentation.

Fold Number	Prostate		Breast	
	Precision	Sensitivity	Precision	Sensitivity
1	86.67	96.05	83.95	94.84
2	87.01	94.92	86.18	95.26
3	91.22	91.33	81.14	96.05
4	93.01	91.37	81.87	96.32
5	85.69	94.85	84.35	96.18
6	94.18	89.28	96.23	76.73
7	96.10	86.81	95.64	85.26
8	93.43	88.31	95.37	84.49
9	92.99	87.74	95.57	85.51
10	93.89	88.12	94.97	89.19
Average	91.42	90.88	89.53	89.98

The F1 scores are 91.15 and 89.75, respectively.

## Data Availability

Not applicable.

## Appendix A

According to the previous research results, we established a skeleton segmentation website equipped with a deep learning framework, enabling clinical physicians to utilize website functions online to assist in calculating BSI and conducting clinical diagnoses, thereby achieving the purpose of the previous research. This website has multiple functions that allow clinical physicians to upload images and perform simple post-processing on the images within the website. Finally, execute the skeleton segmentation deep learning model for skeleton segmentation. The public IP address of the website is 140.128.65.129, and login credentials are required (username: wbbsweb, password: wbbswebpass).
